# Multimodal physiological sensing for the assessment of acute pain

**DOI:** 10.3389/fpain.2023.1150264

**Published:** 2023-06-19

**Authors:** Raul Fernandez Rojas, Niraj Hirachan, Nicholas Brown, Gordon Waddington, Luke Murtagh, Ben Seymour, Roland Goecke

**Affiliations:** ^^1^^ Human-Centred Technology Research Centre, Faculty of Science and Technology, University of Canberra, Canberra, ACT, Australia; ^^2^^ Faculty of Health, Queensland University of Technology, Brisbane, QLD, Australia; ^^3^^ Australian Institute of Sport, Canberra, ACT, Australia; ^^4^^ University of Canberra Research Institute for Sport and Exercise (UCRISE), University of Canberra, Canberra, ACT, Australia; ^^5^^ Department of Anaesthesia, Pain and Perioperative Medicine, The Canberra Hospital, Canberra, ACT, Australia; ^^6^^ Wellcome Centre for Integrative Neuroimaging, University of Oxford, John Radcliffe Hospital, Headington, UK; ^^7^^ Oxford Institute for Biomedical Engineering, University of Oxford, Headington, UK

**Keywords:** Pain, data fusion, physiology, machine learning, non-verbal, EDA

## Abstract

Pain assessment is a challenging task encountered by clinicians. In clinical settings, patients’ self-report is considered the gold standard in pain assessment. However, patients who are unable to self-report pain are at a higher risk of undiagnosed pain. In the present study, we explore the use of multiple sensing technologies to monitor physiological changes that can be used as a proxy for objective measurement of acute pain. Electrodermal activity (EDA), photoplethysmography (PPG), and respiration (RESP) signals were collected from 22 participants under two pain intensities (low and high) and on two different anatomical locations (forearm and hand). Three machine learning models were implemented, including support vector machines (SVM), decision trees (DT), and linear discriminant analysis (LDA) for the identification of pain. Various pain scenarios were investigated, identification of pain (no pain, pain), multiclass (no pain, low pain, high pain), and identification of pain location (forearm, hand). Reference classification results from individual sensors and from all sensors together were obtained. After feature selection, results showed that EDA was the most informative sensor in the three pain conditions, 93.2±8% in identification of pain, 68.9±10% in the multiclass problem, and 56.0±8% for the identification of pain location. These results identify EDA as the superior sensor in our experimental conditions. Future work is required to validate the obtained features to improve its feasibility in more realistic scenarios. Finally, this study proposes EDA as a candidate to design a tool that can assist clinicians in the assessment of acute pain of nonverbal patients.

## 1. Introduction

Pain assessment is one of the most challenging tasks encountered by clinicians (1). This becomes particularly problematic, when clinicians need to assess pain in patients who are unable to communicate (i.e., nonverbal patients). Although pain is considered a subjective experience needing the patients to self-report, the inability to report pain does not negate the possibility that the patient is experiencing pain and in need of pain-reliving treatment (2). In clinical settings, the most reliable method of pain evaluation is the patient’s self-report. This method relies in the patient’s ability to communicate a self-assessment of pain, thus, clinicians can use this information to understand the patient’s pain experience and for pain management. However, patients are often unable to self-report pain due to, for example, being unconscious, sedated, recovering from a stroke, in mechanical ventilation, or suffering from advanced dementia.

When a patient is unable to provide a self-report of pain, other methods for pain assessment are available (3). Pain measurement tools that can be utilised to screen pain in non-verbal patients includes, the behavioral pain scale (BPS), critical-care pain observation tool (CPOT), and the nonverbal pain scale (NVPS). These techniques require highly skilled observers to measure pain (4). Thus, a clear disadvantage of these screening tools is that they are susceptible to assessment bias or misinterpretations, which leads to low inter-rater reliability. Another limitation of these tools is the fact that they often provide a one-time measurement. When multiple observations are constantly needed, the number of clinical observations are labour intensive, which can cause increased workload and nurse burnout (5). These limitations are important to be considered, since they can lead to under-treated pain and unnecessary suffering, or over-treated pain due to strong analgesics and/or analgesic overuse (6).

An important role of clinicians is pain assessment for acute postoperative pain control. It has been reported that three quarters of patients undergoing surgical procedures experience acute pain (7). Acute pain is a type of pain that typically lasts for a short period of time and is usually originated as a response to an injury, illness, or tissue damage. In this context, postoperative pain management is an essential component in facilitating a patient’s recovery to normal function, improve patient comfort, and prevent further complications. It is well documented that inadequate pain assessment and management is associated with increased morbidity and mortality rates (8). In addition, ineffective management of acute pain can cause both physical and psychological distress, such as, depression, anxiety, or chronic pain; it can also lead to an increased risk of complications and, thus, longer hospital stays. Health problems that can lead to increased health care costs for the patient and the public. Therefore, a tool that can assist clinicians in the assessment of acute pain is needed, which will contribute to a more objective, valid, and reliable diagnosis of pain.

Different physiological signals can be used to measure (minimally invasive or noninvasive) physiological changes during acute pain. In the event of acute pain, the autonomic nervous system (ANS) simultaneously affects the function of multiple physiological activities in the body (4). Therefore, sensing technologies that can afford a measure into the ANS are often used. Examples of these technologies include, electrodermal activity (EDA), photoplethysmography (PPG), or respiration (RESP). For example, EDA can capture sympathetic changes related to pain, as it measures the sweat glad activity as response to pain (9). PPG can be used to measure the autonomic response through analysis of heart rate variability (HRV) (10). The respiratory system is also affected by a sympathetic nerves, which have a stimulating effect by increasing oxygen intake in the event of acute pain (11). Although, the use of these sensing technologies can be used individually, multimodality systems have also shown promising results (12–14).

Machine learning has been fundamental for the success of physiological-based systems for the detection of pain. In this context, machine learning is used to better interpret the complexity of pain by revealing patterns within the physiological data. The literature presents different attempts to use multimodal sensing and machine learning for the detection of pain. For instance, in a study by Teichmann et al. (15), ECG and PPG sensors were used to monitor physiological signals from participants and to design a random forest (RF) classifier to identify pain sensations. In another study, Chu et al. (16) used PPG, ECG, and EDA signals to predict the absence and presence of pain using linear discriminant analysis (LDA), k-nearest neighbour (KNN), and support vector machines (SVM). Similarly, Jiang et al. (14) employed EDA, heart rate (HR), breath rate (BR), EDA, and electromyogram (EMG) sensors to identify pain using artificial neural networks. In a study by Yang et al. (17) to investigate the feasibility to use multiple physiological measures to identify pain, a logistic regression model was implemented using oxygen saturation (SpO2), blood pressure (BP), HR, respiration (RESP), and skin temperature data. These examples show that physiological indicators and machine learning can be used in the identification of human pain.

In this study, we explore different pain estimation modalities using multiple physiological sensors (EDA, PPG, RESP). First, we investigate the use of these sensing technologies to identify acute pain (e.g., no pain vs pain). Second, we also explore if pain intensity can be identified in a multiclass problem (e.g., no pain, low pain, high pain). Third, we address the problem of identifying the source of pain using physiological sensors. Consequently, we have designed a stimulation paradigm which affords two intensities (low and high) in two different anatomical locations (forearm and hand). We aim to identify physiological indicators that can be used to trigger technological support to assist clinicians in the assessment of acute pain, in our future work. Therefore, this study presents the following contributions: (1) it offers an exploratory study that aims to compare different sensors technologies in a unimodal and multimodal approach for the objective assessment of acute pain; (2) with a novel dataset, this study serves as a validation of EDA as the superior sensor technology for pain assessment and a possible candidate for further investigation in clinical settings; (3) it presents a set of EDA-based features as potential indicators of acute pain using EDA and machine learning; and (4) to the best of our knowledge, this is the first study that investigates the use of physiological signals to identify the anatomical location where pain originates, which sets baseline results for future studies within the same research stream. The current clinical applications of the proposed are constrained, necessitating further experimentation before its deployment in clinical settings or real-world scenarios can be considered viable.

## 2. Methodology

### 2.1. Participants

Twenty-two participants (12F/10M) took part in the experiment. Their age ranged from 19 to 36 year old (mean age 27±4.19 std). No participants reported a prior history of neurological or psychiatric disorder, a current unstable medical condition, chronic pain, regularly taking medications or being under medication at the time of testing. Participants were given a detailed explanation of the experimental procedures upon their arrival. Written informed consent was obtained before the start of the experiment. The experimental procedures involving human subjects described in this paper were approved by the University of Canberra’s Human Ethics Committee (number:11837).

### 2.2. Experimental procedure

All experiments were conducted at the Human-Machine Interface Laboratory at University of Canberra, Australia. The participants were seated on a chair in front of a table and with both arms resting on the table. The electrodermal activity (EDA) and photoplethysmography (PPG) sensors were placed on the left hand, while the respiration (RESP) sensor was placed on the chest of the participants; all sensors were made by Biosignal plux (Lisbon, Portugal). The two electrodes of the EDA sensor were placed on the proximal phalanx of the index and middle fingers of the hand, and the PPG finger clip sensor was placed on the middle finger. On the right arm, the electrodes of a transcutaneous electrical nerve stimulation (TENS) machine (Medihightec Medical CO., LTD., Taiwan) were placed on the inner forearm and on the back of the hand. These two anatomical locations were used to explore the possibility to identify the source of pain. The location and intensity of pain stimulus were counterbalanced to avoid habituation to repeated experimental pain and to avoid confounding factors due to order effects (18).

**Figure 1:**
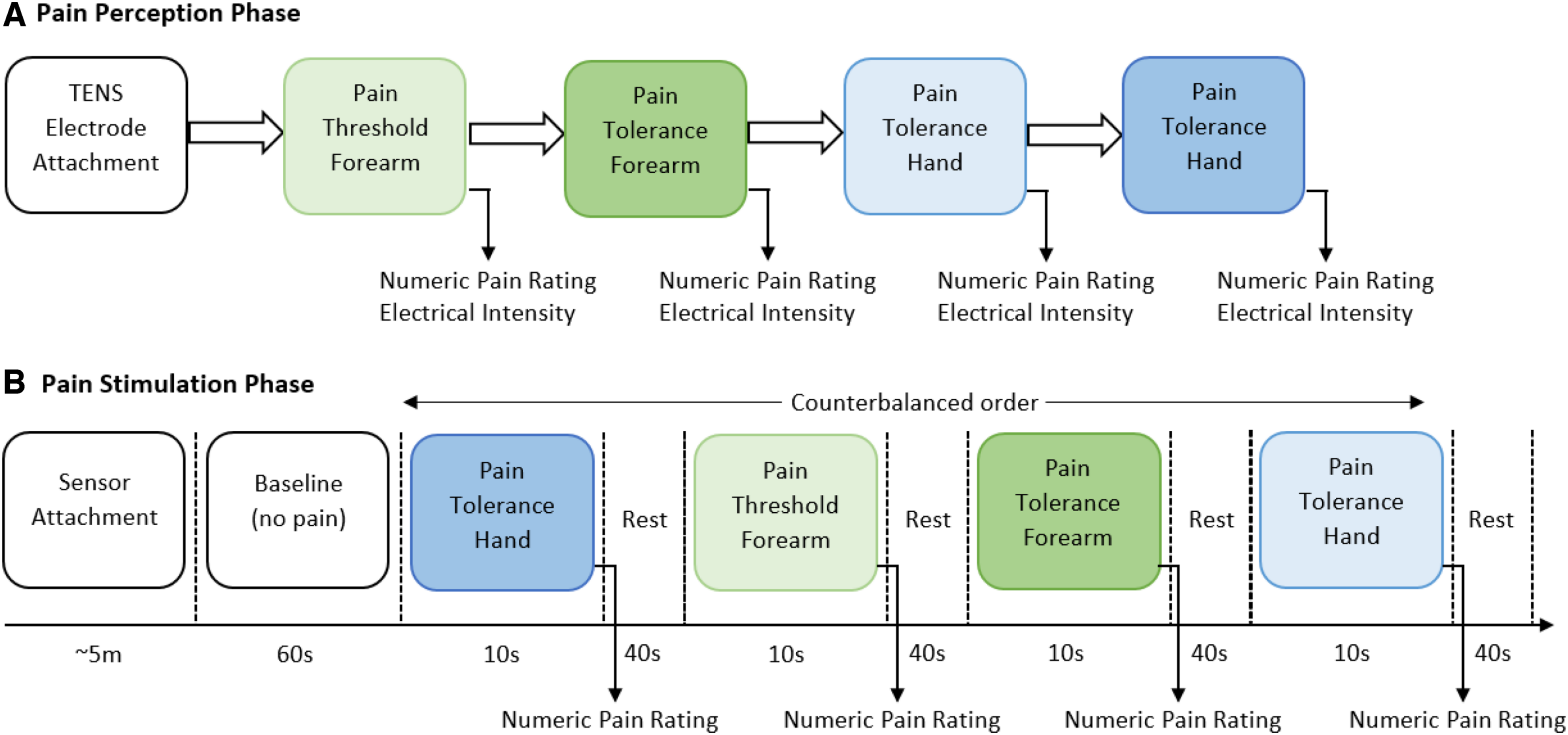
Graphical representation of the two parts of the experimental procedure. (**A**) In the first part of the experiment, individual pain perceptions are obtained. Two anatomical regions (forearm and hand) were used to stimulate participants, a random order was carried out to select the anatomical region. At each location, threshold of pain was first obtained and then pain tolerance was recorded. (**B**) In the second part of the experiment, a randomised order of pain intensity and anatomical location was carried out. Sensor data was recorded throughout this part of the experiment. After each stimulus the individual’s self-reported numeric pain rating was obtained.

The experiment consisted of two main parts, the identification of individual pain perceptions and the pain stimulation part. Figure 1 presents a schematic representation of the experimental procedure. In the first part of the experiment, pain perceptions were obtained using the quantitative sensory testing (QST) protocol (19); no sensors were used in this part of the experiment. The QST protocol is used determine individual’s pain threshold and pain tolerance. We defined pain threshold (low pain) as the lowest stimulus intensity at which stimulation becomes painful, and pain tolerance (high pain) as the highest intensity of pain a person can endure before reaching a point of intolerable discomfort. The participants were exposed to gradually increasing stimulus and were instructed to verbally rate (0 = “no pain,” 10 = “maximum pain”) the pain intensity when the stimulation became painful (pain threshold) and then when the stimulation reached a point where it could no longer be endured (pain tolerance). The intensity of the TENS machine, in which the threshold and tolerance of pain occurred, were recorded to be used as the intensity during the stimulation part.

In the second part, the pain intensity and anatomical location was studied. Before the start of the pain stimulation sequence, the physiological sensors were placed on the hand (EDA and PPG) and the chest (RESP) of the participant. At the start of the experiment, a 60-second baseline period was recorded, in which the participants were instructed to remain calm; this baseline period served as the no-pain condition for the classification tasks. After that, the counterbalanced design alternated stimuli intensity (low or high) and location (forearm or hand). Six repetitions with a duration of 10 s for each stimulus were recorded. Immediately after each stimulus, the participants were asked to verbally rate the stimulation using the same scale (0 = no pain, 10 = maximum pain) used during the pain perception. After rating the pain intensity, a 40-second rest period was offered to allow all physiological signals to return to baseline. In total, twenty-four recordings (2 intensities × 2 locations × 6 repetitions) were obtained from each participant. Each experiments lasted for approximately one hour (30 min preparation and individual pain perception, and 30 min pain stimulation experiment).

### 2.3. Pre-processing

All physiological data was sampled at 100 Hz. Each physiological signal was treated separately to remove noise. The respiration data were filtered with a band-pass Butterworth filter (0.1–0.5 Hz), as this frequency range corresponds to the respiratory frequency of the human body (20). For the EDA data, a low-pass Butterworth filter (5 Hz) was applied to remove high-frequency oscillation and power line interference. Then, the EDA signals were separated into their phasic and the tonic components following the standard procedure by Makowski et al. (21), with a high-pass (0.05 Hz) and low-pass (0.05 Hz) Butterworth filter, respectively; however, the phasic component was chosen for the remaining of the analysis due to it provides better response to the rapid changes in electrodermal activity in response to the stimuli. The PPG data were filtered with a band-pass Butterworth filter (0.04–1.7 Hz) to obtain cardiovascular-derived information corresponding to the normal heart beat (60–100 bps) and respiratory sinus arrhythmia (22).

### 2.4. Data analysis

#### 2.4.1. Validation of experimental conditions

The study used a within-subjects design, in which each participant completed all the experimental conditions. The four experimental conditions were: (1) low pain on hand, (2) low pain on forearm, (3) high pain on hand, and 4) high pain on forearm. To validate the design of the experiment and the experimental conditions, pain ratings were self-reported by the participants after each stimulus were used as the criterion measure of pain. Normality of data distribution was checked with Shapiro-Wilk test. This test showed that data did not significantly deviate from a normal distribution (p>0.05), thus, parametric testing was used. A one-way analysis of variance (ANOVA) was conducted (with p<0.05 considered statistically significant) to determine if the pain ratings were related to the experimental conditions (High and Low pain) across all the participants. Thus, the null hypotheses (Ho) is that the perceived pain is not due to the experimental condition. When significant differences were identified, post-hoc comparisons were carried out using Tukey’s HSD (honestly significant difference) test.

#### 2.4.2. Feature extraction

In order to extract features from the sensor data, all sensor data was analysed using windows. A 10-second window size was used for feature extraction, as this window size was the optimal after testing several window sizes (e.g., 1–10 s) with the three sensors combined. Two types of features were obtained from each window of data, these are: statistical features from all physiological sensors and specialised features from individual sensors (EDA, RESP, PPG). From all three physiological sensors, 10 well-known statistical feature were extracted (23), including: Mean, standard deviation, Min, Max, Range, Median, Sum, Range, Q1, Q2, Q3, Q4, and Inter-Quartile Range. Specialised features were obtained using the Neurokit2 Python toolbox (21). For the complete set of extracted features, the interested reader is referred to the cited reference for detailed description. After removing features with missing values greater than 50% of the total number of samples, a total of 122 features were obtained. The number of extracted features from the different sensors was: 12 features from EDA, 30 from RESP, and 80 from PPG. Table 1 presents a summary of the extracted features. The range of each feature was re-scaled to a [0,1] range to standardised the range of features.

**Table 1:** Summary of features obtained during the feature extraction process.

Sensor	Features
EDA	Phasic max peak amplitude, number of EDA peaks, and amplitude measures (mean, median, standard deviation, max, min, range and interquartile range metrics)
RESP	Respiratory rate variability indices, respiratory sinus arrhythmia indices, RESP amplitude measures (min, max, mean), and RESP rate changes (min, mean, max, time of min, time of max)
PPG	PPG rate characteristics (mean, amplitude) and heart rate variability (HRV) indices (time, frequency, and nonlinear domain)

#### 2.4.3. Feature selection

Feature selection was performed to reduce the number of features and obtain a more accurate learning model. Feature selection was carried out after the feature extraction process. The selection criteria was based on joint mutual information (JMI), this method can be used to rank the features according to their cumulative summation of the mutual information (24). JMI aims to identify the most informative features that have a high degree of relevance to the target variable while minimising the redundancy between features themselves. JMI measures the mutual information between a target variable and each feature to quantify the level of statistical dependency between variables, i.e., it measures the amount of information that each feature provides about the target variable. Features with high mutual information are considered to be more informative and are more likely to contribute to accurate predictions. In addition, this method employs the mutual information among the features themselves to identify redundant or irrelevant features. In this way, JMI captures unique and diverse information from a set of features, by identifying features that have high MI with the target variable and low MI with each other. The reason JMI was chosen is because it presents a good trade-off in terms of accuracy, stability, and flexibility than other ranking methods (25). Additionally, JMI evaluates the features independently of any classification model and it is computed only once (26). A feature fusion approach was followed to concatenate all computed features before the classification task.

#### 2.4.4. Classification

The main objective of the classification was to identify the pain intensity under various pain conditions using the physiological data. Three main classification tasks were performed, these are: no pain vs pain (high pain), no pain vs low pain vs high pain (3-class problem), and pain on forearm (low and high) vs pain on hand (low and high). The dataset was composed with the same number of samples (six windows of 10 s) in each class, in total 528 samples (6 repeats × 2 pain intensities × 2 locations × 22 subjects) were obtained. Three well-known machine learning models were use to perform the classification, including linear discriminant analysis (LDA), decision trees (DT), and support vector machines (SVM) using the Gaussian Kernel (RBF). All learning models and classification tasks were implemented using Python 3.10.

**Figure 2:**
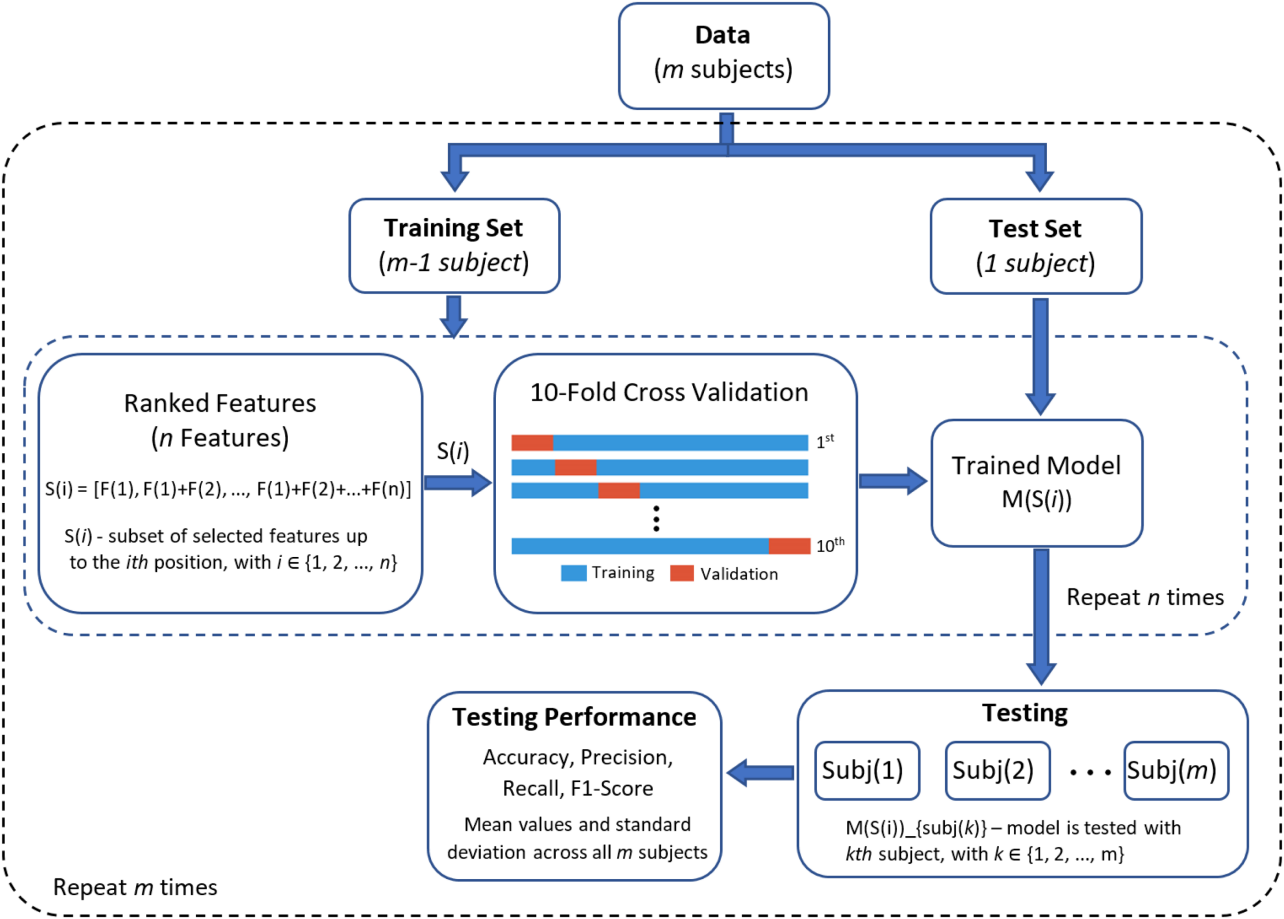
Graphical representation of the classification process using leave-one-subject-out cross validation (LOSO-CV) for performance evaluation.

A leave-one-subject-out cross validation was implemented to evaluate the classifiers. In this method of cross validation, the learning models are trained with the data of 21 participants and then tested with the data of the remaining participant. This method is repeated until all of the subjects had been part of the testing dataset. First, reference performance values were obtained using all features (i.e., without feature selection) from each sensor separately and then with all sensors. In this step, parameter optimisation of learning models was carried out using a grid search with a 10-fold cross validation, using the training set only and with all 122 features. These parameters were: for SVM, C [1,10,100,100] and γ [0.0001,0.001,0.01,0.1] parameters; for DT, the criterion function [Gini, Entropy]; and for LDA, no parameters were optimised as LDA presents a closed-form solution (27). Second, after feature selection using JMI, all features were ranked and used to train and test the classifiers. Figure 2 presents the graphical representation of this process. In both cases, the final generalisation results are presented as the average value and standard deviation of performance metrics across all subjects in the testing phase.

## 3. Results

**Figure 3:**
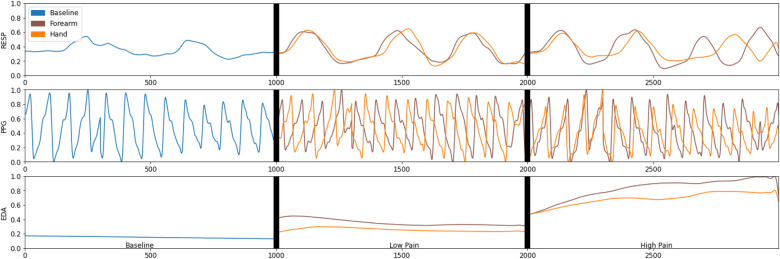
An example of the physiological data captured by the electrodermal activity (EDA), respiration (Resp), and photoplethysmography (PPG) during the different experimental conditions.

In this section, the results of the empirical comparison between the learning models (LDA, SVM, DT) to identify the different experimental conditions are presented. The analysis was carried out in three main parts. First, the results of the validation of the experimental conditions are presented. Second, reference classification results were obtained from each individual sensor and all together. Third, feature optimisation is implemented to identify the best feature set to classify each pain condition. Figure 3 presents an example of the physiological response obtained from all three sensors after each stimulus. It is evident that the EDA sensor presents a clear distinction between the different experimental conditions.

### 3.1. Validation of experimental conditions

**Figure 4:**
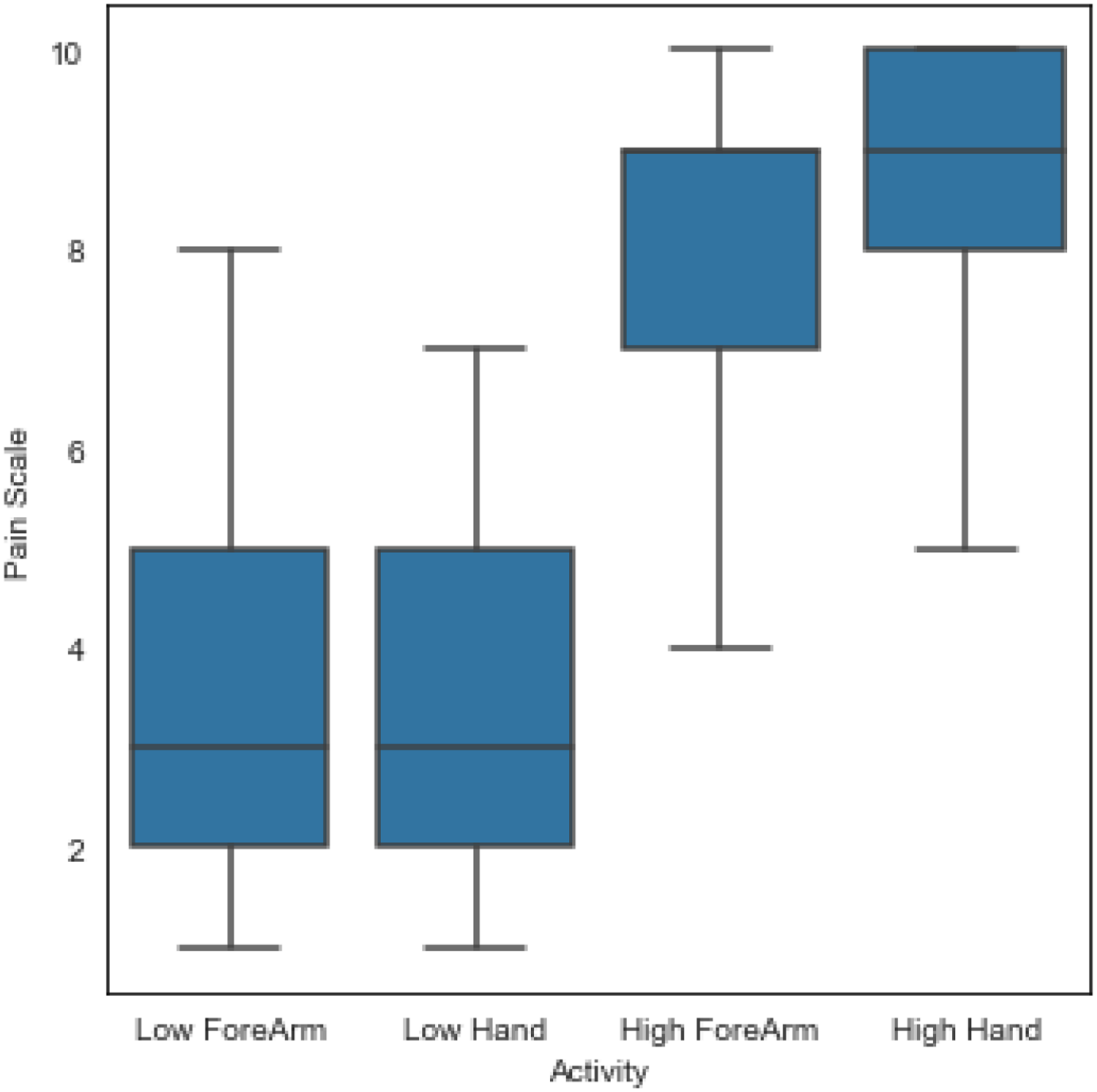
A summary of all individual pain ratings based on the four experimental conditions.

First, a summary of the individual pain ratings based on the four experimental conditions is presented in Figure 4. It is clear that pain tolerance (high pain) was rated higher than pain threshold (low pain) as the pain tolerance represents a much higher stimulation intensity. In the design of the experimental conditions, it was expected that the higher the stimulation intensity, the higher the participant’s pain experience and thus, the higher the pain rating; and the participant’s pain rating followed the expected response. In terms of anatomical location and intensity of pain, it is evident that the numeric pain rating during the tolerance of pain (high pain) condition was slightly higher when the stimulation was delivered on the hand (8.69±1.16) than on the forearm (8.37±1.34). Similarly, the numeric pain rating during the threshold of pain (low pain) condition was slightly higher when the stimulation was applied on the hand (3.45±1.57) than on the forearm (3.31±1.65).

Second, to validate the experimental conditions the numeric pain rating from all participants were analysed using statistical methods described above. There was a statistically significant difference in pain ratings between at least two groups (F(2,18)=401.23, p=0.000). Tukey’s HSD test for multiple comparisons found that the mean value of pain ratings was significantly different between pain tolerance (high pain) and pain threshold (low pain) in both, arm (p<0.001, 95% C.I.=[−5.78,−4.69]) and forearm (p<0.001, 95% C.I.=[−5.59,−4.51]). There was no statistically significant difference in mean pain ratings between the two locations of stimulation (arm and forearm) during pain tolerance (high pain) (p=0.413, 95% C.I.=[−0.21,0.86]) and during pain threshold (low pain) (p=0.91, 95% C.I.=[−0.40,0.68]). A summary of descriptive statistics is presented in Table 2. Additional comparisons between the two anatomical locations and the two different intensities (e.g., High Arm vs Low Forearm) are also presented in the table. The confidence interval (C.I.) are narrow, suggesting that our estimate of the difference is relatively precise. This reflects that the experimental conditions did affect the pain ratings and that the number of participants did not affect the precision of our estimates in this study.

**Table 2:** Descriptive statistics showing the results of the one-way ANOVA test along with the Tukey’s HSD post-hoc multiple comparisons test.

Group 1	Group 2	Mean diff.	p-adj	Lower	Upper	Reject *Ho*
High arm	High hand	0.3241	0.413	−0.2178	0.8659	False
High arm	Low hand	−5.0556	**0.000**	−5.5974	−4.5137	True
High arm	Low hand	−4.9167	**0.000**	−5.4585	−4.3748	True
High hand	Low arm	−5.3796	**0.000**	−5.9215	−4.8378	True
High hand	Low hand	−5.2407	**0.000**	−5.7826	−4.6989	True
Low arm	Low hand	0.1389	0.911	−0.403	0.6808	False

### 3.2. Reference values

Based on the computed features from each sensor, reference performance results are obtained. The performance of the learning models are investigated with features from each sensor separately and then with all features combined. The results on the test set using LDA, SVM, and DT are presented in Table 3. The results represent the classification accuracy and standard deviation, while the number of features used in each model appears in parenthesis. The reference performance results will be used to evaluate the learning models during the feature selection process. Three classification tasks were investigated: identification of pain (no pain, high pain), multiclass (no pain, low pain, high pain), identification of pain location (forearm, arm).

**Table 3:** Reference accuracy results obtained with different features from each sensor and in combination, the number of features appears in parentheses.

Task	Model	PPG (80)	EDA (12)	RESP (30)	ALL (122)
	LDA	81.1±14	92.8±7	71.6±6	89.5±13
No Pain, Pain	SVM	82.9±11	93.1±10	71.8±4	93.1±10
	DTC	76.7±16	88.5±11	63.4±10	92.0±10
	LDA	53.9±11	64.3±14	44.7±10	66.4±13
Multiclass	SVM	53.2±10	67.8±16	46.2±9	67.2±14
	DTC	47.5±10	59.3±13	38.4±9	56.5±13
	LDA	49.1±5	49.8±8	50.4±8	49.8±6
Pain Location	SVM	50.9±5	47.8±6	51.3±8	51.4±8
	DTC	53.1±10	49.9±8	50.3±6	48.6±7

Classification results for the three learning tasks presented various results. First, EDA data produced the best results for the identification of pain (no pain, pain). Two learning models exhibited the best results using twelve features from EDA data, LDA (acc=0.938) and SVM (acc=0.931). On the other hand, performance results using a combination of all features (n=122) exhibited similar results using the SVM model (acc=0.931). Second, EDA also exhibited the best results in the multiclass task. In this case, SVM produced the highest results with EDA alone (acc=0.678) and a similar response with data from all sensors combined (acc=0.672). Third, PPG features presented the best results for the identification of pain location (forearm, hand). DT produced the best result (acc=0.531) with features only from PPG data.

### 3.3. Classification after feature selection

**Figure 5:**
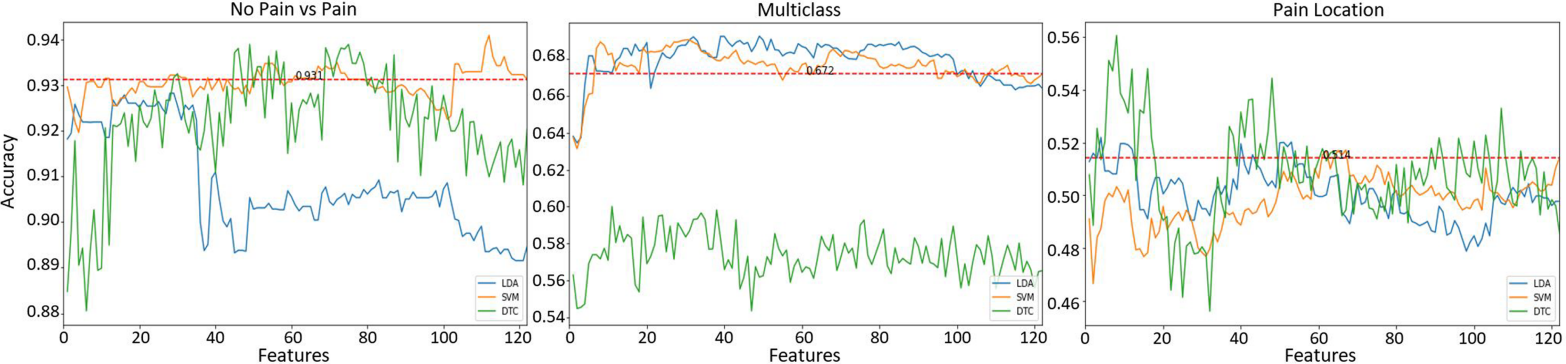
Classification results after feature selection of the different learning tasks. Horizontal red (dashed) line represents the best accuracy result using all (n=122) features without feature selection.

After using joint mutual information (JMI) to find the significance of each feature, all features were ranked and used to train and test the classifiers (refer to Figure 2). The identification of the best performing model was systematically tested with a different number of features based on their feature importance. This process of feature evaluation was carried out for each of the classification tasks. Results for individual learning tasks are presented in Figure 5, a systematic search to identify the best feature set was implemented using three learning models: SVM (orange line), LDA (blue line), and DT (green line). The results of each individual learning task are presented in the following sections.

#### 3.3.1. Identification of pain

The first learning task to be evaluated was the identification of pain (No pain vs High Pain). Figure 5 (left panel) presents the classification results. The average accuracy results (acc=93.1) using all features (n=122) together is presented as a horizontal red line (refer to Table 3) as a reference value. The best result (acc=94.1±8) was obtained using the SVM model with 112 features, the DT model exhibited comparable results (acc=93.8±7) with 49 features, and the LDA model presented slightly lower results (acc=92.8±9) with 28 features. It is clear that SVM and DT models obtained better results than the reference results using less features. In addition, it is possible to observe that using only 10 features, the SVM model presents comparable results (acc=93.2±8) than the reference value (acc=93.1). This set of 10 features is composed only by EDA-based features, these are (by decreasing order of importance): EDA_range, EDA_std, EDA_max, EDA_q3, EDA_sum, EDA_mean, EDA_median, EDA_q1, EDA_lqr, EDA_min.

#### 3.3.2. Multiclass problem

A multiclass learning problem (No pain, Low Pain, High Pain) was the second learning task. Figure 5 (middle panel) presents the results for the multiclass learning problem. Two models (SVM and LDA) presented better results than the reference accuracy result (acc=67.2±14) using a combination of all obtained features. Overall, the best result (acc=69.2±14) was obtained using LDA with 49 features. Comparable results (acc=69.0±16) were obtained with the SVM classifier using 30 features. The worst performance out of the three learning models was exhibited by the DTC model, the best performance (acc=60.0±13) of the DTC model was obtained using 11 features. Again, it is possible to observe that the SVM classifier using only 8 features produced slightly lower results (acc=68.9±14) than the best results obtained by the LDA model; these 8 features also exhibited better results than the reference value (acc=67.2±14). This set of 8 features is also composed only by EDA-based features, including (by decreasing order of importance): EDA_max, EDA_q3, EDA_median, EDA_q1, EDA_mean, EDA_sum, EDA_range, EDA_min.

#### 3.3.3. Pain location

The last learning task was the identification of pain location, i.e., identify the anatomical location where pain originates. Two anatomical locations were investigated, the forearm and the hand, both on the participant’s right arm. Figure 5 (right panel) presents the classification results for this learning task. Again, reference values (acc=51.4±8) using all available features is presented as a red dashed line. In this case, all three models exhibited better results that the reference value. The SVM model presented the lowest result (acc=51.7±5) out of the three classifiers, this model used 67 features. The second best result (acc=52.1±10) was obtained by the LDA model with only 4 features. Finally, the best accuracy result (acc=56.0±8) was obtained with the DT using 8 features. This set of 8 features are only composed of features from EDA data, these are (by decreasing order of importance): EDA_q3, EDA_max, EDA_min, EDA_q1, EDA_median, EDA_lqr, EDA_mean, EDA_sum. Table 4 presents a summary of the accuracy results obtained with the best feature subset for each classification task.

**Table 4:** Summary of results (including ±std) obtained in this study from the three learning tasks after feature selection.

Task	Model	Sensor	Features	Accuracy	Precision	Recall	F1-Score
No Pain vs Pain	SVM	EDA	10	93.2±8	94.6±6	96.8±3	95.5±3
Multiclass	SVM	EDA	8	68.9±14	70.8±8	68.9±8	68.5±8
Pain location	DT	EDA	8	56.0±8	55.2±6	56.4±13	55.4±7

## 4. Discussion

In this study, we have carried out a stimulation experiment with the aim to identify a set of indicators that can be used to assess human pain. An experiment was designed to stimulate participants with two intensity levels (low and high) and at two different anatomical locations (forearm and hand). A total of 112 features were computed from three different sensors (EDA, PPG, and Resp). Using a feature selection technique, the most important features were obtained for each learning task, and then these features were evaluated during the learning tasks. After each classification tasks, a more compact set of features were identified. This set of features represent a group of possible physiological indicators for the objective assessment of pain.

The experimental conditions and overall assumption of the experiment were validated. An increase of stimulation intensity led to an increase in the participant’s perceived pain experience (p<0.000). This is in line with previous experiments carried out in other studies (9, 18). For instance, in an experiment to observe the effect of thermal pain stimulation on EDA data (9), it was found statistical significant differences between their no pain and pain conditions. Similarly, in a study using functional near-infrared spectroscopy (fNIRS) and thermal stimulation (18), significant higher individual’s numeric pain ratings were identified during pain tolerance than during pain threshold stimuli.

This study presents promising results for the identification of pain (i.e., no pain vs pain) using EDA sensor data. Although, the best results were obtained with 112 features from a combination of all three sensors (EDA, RESP, PPG) with an accuracy of 94.1%, it was observed that the SVM model obtained comparable results with the top-10 ranked features with an accuracy of 93.2%. It is worth mentioning that this set of 10 features are computed from EDA data, which presented the highest weights in the context of a multimodal classifier. Direct comparisons with other studies are difficult because of the use of different experimental conditions, biosignals, sampled populations and with different demographics, validation methods, and classification models (28). In comparison with other studies for the identification of pain using EDA sensor data, our study has exhibited comparable results. For instance, Susam et al. (29) used EDA to discriminate between high pain (moderate to severe) versus no pain in children’s postoperative pain, with an accuracy of 77.66% using linear SVM. In another study using EDA data collected from postoperative patients, Aqajari et al. (30) used a random forest (RF) model to discriminate between baseline (no pain) and low pain, and between baseline and high pain with an accuracy of 86% and 61.5%, respectively. In another study to identify pain using the EDA response in experimental thermal pain, Kong et al. (9) used a polynomial SVM learning model to discriminate between no pain and pain with an accuracy of 90%. In another study by Kong et al. (31), an accuracy of 87% was obtained using a Gaussian SVM to identify between no pain and high pain using EDA data. Table 5 presents a summary from other studies using EDA.

**Table 5:** Comparable results from studies using EDA data to identify acute pain (No Pain vs Pain).

Study	Sensor	Pain stimulus	Model	Accuracy
Susam et al. (29)	EDA	Postoperative	L-SVM	77.6%
Aqajari et al. (30)	EDA	Postoperative	RF	61.5%
Kong et al. (9)	EDA	Thermal	P-SVM	90%
Kong et al. (31)	EDA	Electrical	G-SVM	87%
This study	EDA	Electrical	G-SVM	93.2%

In the multiclass problem, we were able to classify three different pain levels (No Pain, Low Pain, High Pain), but the accuracy was relatively lower than the binary outcomes (No Pain, Pain). Although, it was expected as the baseline accuracy in the three-class classification problem would be 33.33% or 1/3, another possible reason for obtaining a lower accuracy is the common similarities between the physiological response among the three experimental conditions; this can be observed in Figure 3. The obtained accuracy is in line with other studies trying to identify multiple pain conditions in a multiclass problem. For instance, in a study by Kong et al. (31) to investigate the use of EDA sensor data to identify pain, a Gaussian SVM was used to identify between low, medium, and high pain with an accuracy of 63%. In another study, Jiang et al. (14) used heart rate (HR), breath rate (BR), EDA, and facial electromyogram (EMG) to identify between No Pain, Mild Pain, and Severe Pain with an accuracy of 68.2% using artificial neural networks. Yang et al. (17) used blood oxygenation (SpO2), blood pressure (BP), HR, RESP, and temperature (TEMP) to identify pain (baseline, low pain, and high pain) in patients with sickle cell disease with an accuracy of 40.4% using a multinomial logistic regression model.

This study also explored the possibility to identify the source of pain using physiological data. The classification result exhibited the lowest accuracy (acc=56.0%) among the three learning tasks, which it was expected as this task is extremely difficult; in particular, as the stimulation sites were on the same arm. To the best of our knowledge there is no other study that explores the possibility to identify the location of pain using physiological data. The need for neural sensors (e.g., EEG, fNIRS) that provide additional information would make pain localisation more accurate (9). The idea to find the source of pain is important for patients unable to communicate (i.e., non-verbal patients). Particularly useful with the current COVID-19 pandemic, especially for intubated patients (32). As being intubated can be painful and traumatic even with administration of sedatives and analgesics. In a study by Clukey et al. (33), it was found that sedation may mask uncontrolled pain for intubated patients and prevent them from communicating this condition to a nurse. When the intubated patient is conscious, the patient can indicate the location of their pain by pointing to the site or to the location on a body picture. Unfortunately, when the intubated patient is not able to self-report, pain localisation is extremely difficult. In these cases, nurses follow protocols for pain screening that include a physical examination using palpation and auscultation to determine pain and pain localisation. Nurses look for non-verbal indicators while conducting the examination, including, crying, whining, facial expressions, or protective body movement as guarding or clutching a body part. However, this is a complex task and sometimes misinterpretation of information can occur. Therefore, a system that is able to identify pain and the pain location can be extremely helpful for nurses to assess pain in non-verbal patients.

A multimodal approach (EDA, PPG, RESP) was designed in this study, however, EDA sensor data exhibited the best classification results in all the learning tasks. The use of multiple sensing technologies was used with the expectation that improvements in classification accuracy would be obtained. Although, this was partially true because the combined features (n=120) from all sensors exhibited, in some of of the classification tasks, slightly better results than individual sensors. However, after the feature selection process, EDA-based features showed better accuracy than the other sensing technologies. This is in line with different studies using multimodal sensor systems, in particular with studies using the Biovid and X-ITE datasets. For instance, in an study using EDA, electrocardiogram (ECG), and electromyogram (EMG) showed that EDA-based features presented better classification results to discriminate between baseline (no pain) and pain tolerance (high pain) (34). In another experiment using EDA, ECG, and EMG, it was found that EDA was the most information rich sensor for continuous pain intensity prediction (35). Similarly, EDA was found to be the best single modality in both classification and regression using EDA, ECG, and EMG sensors (36). In our study, the EDA-based features represents a more compact set of features that can produce less complex learning models. An advantage of finding the best sensor modality is that EDA affords data collection from a smartwatch or wrist biosensor, which can be easy to collect for long periods of time, in different conditions (e.g., during transportation within the hospital, or at night when the patient is asleep), remote monitoring (e.g., at home), and without being obtrusive to the patient.

All physiological signals (EDA, PPG, RESP) investigated in this study are closely related to the autonomous nervous system (ANS). The autonomic system is related to the regulation of involuntary, physiological processes such as regulating sweating, blood pressure, or respiration. The ANS is also linked to pain as painful stimuli trigger an autonomic defensive response in the body, this response is designed to prevent further harm and facilitate escape from the painful stimuli (37). A key element to the immediate response to pain in the ANS is the sympathetic nervous system (SNS), which is responsible for the body’s fight or flight response. When experiencing pain, the SNS initiates a physiological response that results in various changes such as alterations in blood pressure, oxygen consumption, and sweating (38). When SNS is activated due to pain EDA can be used as measure of SNS activity since increased SNS activity can lead to an increase in sweat (i.e., an increase in electrical conductance on the skin). Similarly, PPG and RESP can assess sympathetic activity by measuring changes in skin blood flow and respiration rate, respectively. However, PPG and RESP are also influenced by other factors, such as temperature and hydration in PPG, and anxiety or physical activity in RESP data. On the other hand, EDA is considered a relatively direct measure of SNS activity, since changes in skin conductance are primarily regulated by the SNS innervation of sweat glands in the skin (39). Thus, compared to PPG and RESP, EDA may be better suited for detecting pain in terms of SNS activity due to it offers a relatively more direct measure of sympathetic activation.

Preliminary results presented in this study are promising, however, we acknowledge that this study presents some limitations that should be addressed in our future research. Confounding factors such as stress, anxiety, or other emotions can affect the identification of pain, in particular due to anticipation of the upcoming pain stimulation. Therefore, in our future research experiments, we will try to minimise the participants’ stress as much as possible by asking subjects to keep their eyes closed to avoid pain anticipation or by varying the length of rest periods between stimuli (40, 41). In addition, acute pain in clinical contexts may have variable onset dynamics, making the identification of pain more difficult if onset time is unknown. A possible solution will be the use of a moving window classifier to build learning models that identify different onsets of pain. It is also important to mention that further research will be needed for pain detection in chronic pain patients. Another limitation of the current study is the lack of neurophysiological sensors (e.g., fNIRS, EEG), as these type of sensors can improve overall accuracy and also improve our results in the location of pain (42). In our future work, we will also explore more advanced classification techniques (e.g., deep learning) that can help us improve the current results. Finally, it is worth noting that clinical applicability of the current system will need further experimentation and research, including the need to incorporate participants with broader age range and different pain conditions, so the learning model could generalise better to different populations.

## 5. Conclusions

This study investigated multiple sensing technologies to identify different pain estimation scenarios. Three different sensors (EDA, PPG, and RESP) were employed to identify a set of indicators that can be used for the objective assessment of pain. The experimental conditions used in this study were validated by analysing the self-reported participants’ numeric pain rating using statistical analysis. We obtained reference classification results from each individual sensor and from a combination of all sensors together. After a feature selection process, results showed that EDA was the most informative sensor for prediction in the three pain conditions (identification of pain, multiclass, and location of pain). To the best of our knowledge, this is the first study that investigates the use of physiological signals to identify the anatomical location where pain originates, which sets baseline results for future studies within the same research stream. The current clinical applications of the proposed approach highlight the necessity for further experimentation to establish its suitability for deployment in clinical settings or real-world scenarios. Finally, future research will utilize the proposed physiological indicators for further investigation and the development of a tool aimed at assisting clinicians in the assessment of acute pain.

## Data Availability

The data that support the findings of this study are available from RFR, upon reasonable request. Requests to access the datasets should be directed to RFR, raul.fernandezrojas@canberra.edu.au.

## References

[1] McGuire DB Kaiser KS Haisfield-Wolfe ME Iyamu F. Pain assessment in noncommunicative adult palliative care patients. Nurs Clin. (2016) 51:397–431. 10.2196/25258 PMC4978178 27497016

[2] Merskey HE. Classification of chronic pain: descriptions of chronic pain syndromes, definitions of pain terms. Pain. (1986). 3461421

[3] Deldar K Froutan R Ebadi A. Challenges faced by nurses in using pain assessment scale in patients unable to communicate: a qualitative study. BMC Nurs. (2018) 17:1–8. 10.1186/s12912-018-0281-3 29568232 PMC5857143

[4] Subramaniam SD Doss B Chanderasekar LD Madhavan A Rosary AM. Scope of physiological, behavioural pain assessment techniques in children—a review. Healthc Technol Lett. (2018) 5:124–9. 10.1049/htl.2017.0108 30155264 PMC6103781

[5] Roué JM Morag I Haddad WM Gholami B Anand KJ. Using sensor-fusion, machine-learning algorithms to assess acute pain in non-verbal infants: a study protocol. BMJ Open. (2021). 11, e039292. 10.1136/bmjopen-2020-039292 PMC7789448 33408199

[6] Herr K Coyne PJ McCaffery M Manworren R Merkel S. Pain assessment in the patient unable to self-report: position statement with clinical practice recommendations. Pain Manag Nurs. (2011) 12:230–50. 10.1016/j.pmn.2011.10.002 22117755

[7] Köse Tamer L Sucu Dağ G. The assessment of pain and the quality of postoperative pain management in surgical patients. Sage Open. (2020) 10:2158244020924377. 10.1177/2158244020924377

[8] Ayasrah SM O’Neill TM Abdalrahim MS Sutary MM Kharabsheh MS. Pain assessment and management in critically ill intubated patients in Jordan: a prospective study. Int J Health Sci. (2014) 8:287. 10.12816/0023981 PMC4257364 25505864

[9] Kong Y Posada-Quintero HF Chon KH. Pain detection using a smartphone in real time. In *2020 42nd Annual International Conference of the IEEE Engineering in Medicine & Biology Society (EMBC)*. IEEE (2020). p. 4526–9. 10.1109/EMBC44109.2020.9176077 33019000

[10] Choi B-M Yim JY Shin H Noh G. Novel analgesic index for postoperative pain assessment based on a photoplethysmographic spectrogram and convolutional neural network: observational study. J Med Internet Res. (2021) 23:e23920. 10.2196/23920 33533723 PMC7889419

[11] Swift A. Understanding pain, the human body’s response to it. Nurs Times. (2018) 114:22–6.

[12] Webber M Rojas RF. Human activity recognition with accelerometer, gyroscope: a data fusion approach. IEEE Sens J. (2021) 21:16979–89. 10.1109/JSEN.2021.3079883

[13] Hirachan N Mathews A Romero J Rojas RF. Measuring cognitive workload using multimodal sensors. In 2022 44th Annual International Conference of the IEEE Engineering in Medicine & Biology Society (EMBC). Glasgow: IEEE (2022). p. 4921–24. Available from: 10.1109/EMBC48229.2022.9871308. 36085818

[14] Jiang M Mieronkoski R Syrjälä E Anzanpour A Terävä V Rahmani AM, et al. Acute pain intensity monitoring with the classification of multiple physiological parameters. J Clin Monit Comput. (2019) 33:493–507. 10.1007/s10877-018-0174-8 29946994 PMC6499869

[15] Teichmann D Klopp J Hallmann A Schuett K Wolfart S Teichmann M. Detection of acute periodontal pain from physiological signals. Physiol Meas. (2018) 39:095007. 10.1088/1361-6579/aadf0c 30183680

[16] Chu Y Zhao X Han J Su Y. Physiological signal-based method for measurement of pain intensity. Front Neurosci. (2017) 11:279. 10.3389/fnins.2017.00279 28603478 PMC5445136

[17] Yang F Banerjee T Narine K Shah N. Improving pain management in patients with sickle cell disease from physiological measures using machine learning techniques. Smart Health. (2018) 7:48–59. 10.1016/j.smhl.2018.01.002 30906841 PMC6428053

[18] Rojas RF Huang X Ou K-L. Toward a functional near-infrared spectroscopy-based monitoring of pain assessment for nonverbal patients. J Biomed Opt. (2017) 22:106013. 10.1117/1.JBO.22.10.106013 29076307

[19] Rolke R Magerl W Campbell KA Schalber C Caspari S Birklein F, et al. Quantitative sensory testing: a comprehensive protocol for clinical trials. Eur J Pain. (2006) 10:77–88. 10.1016/j.ejpain.2005.02.003 16291301

[20] Nakajima K Tamura T Miike H. Monitoring of heart and respiratory rates by photoplethysmography using a digital filtering technique. Med Eng Phys. (1996) 18:365–72. 10.1016/1350-4533(95)00066-6 8818134

[21] Makowski D Pham T Lau ZJ Brammer JC Lespinasse F Pham H, et al. Neurokit2: a python toolbox for neurophysiological signal processing. Behav Res Methods. (2021) 53:1689–96. 10.3758/s13428-020-01516-y 33528817

[22] Luo S Zhou J Duh HB-L Chen F. BVP feature signal analysis for intelligent user interface. In Proceedings of the 2017 CHI Conference Extended Abstracts on Human Factors in Computing Systems. Denver: Association for Computing Machinery (2017). p. 1861–8.

[23] Fernandez Rojas R Huang X Ou K-L. A machine learning approach for the identification of a biomarker of human pain using fNIRS. Sci Rep. (2019) 9:1–12. 10.1038/s41598-019-42098-w 30948760 PMC6449551

[24] Bennasar M Hicks Y Setchi R. Feature selection using joint mutual information maximisation. Expert Syst Appl. (2015) 42:8520–32. 10.1016/j.eswa.2015.07.007

[25] Brown G Pocock A Zhao M-J Luján M. Conditional likelihood maximisation: a unifying framework for information theoretic feature selection. J Mach Learn Res. (2012) 13:27–66.

[26] Guyon I Elisseeff A. An introduction to feature extraction. Feature extraction: foundations and applications. Vancouver: Springer (2006).

[27] Sifaou H Kammoun A Alouini M-S. High-dimensional linear discriminant analysis classifier for spiked covariance model. J Mach Learn Res. (2020) 21(1):4508–31.

[28] Rojas RF Huang X Romero J Ou K-L. fNIRS approach to pain assessment for non-verbal patients. In *Neural Information Processing: 24th International Conference, ICONIP 2017, Proceedings, Part IV 24*; Nov 14–18; Guangzhou, China. Springer (2017). p. 778–87.

[29] Susam BT Akcakaya M Nezamfar H Diaz D Xu X de Sa VR, et al. Automated pain assessment using electrodermal activity data, machine learning. In *2018 40th Annual International Conference of the IEEE Engineering in Medicine, Biology Society (EMBC)*. IEEE (2018). p. 372–5. 10.1109/EMBC.2018.8512389 PMC6436808 30440413

[30] Aqajari SAH Cao R Naeini EK Calderon M-D Zheng K Dutt N, et al. Pain assessment tool with electrodermal activity for postoperative patients: method validation study. JMIR Mhealth Uhealth. (2021) 9:e25258. 10.2196/25258 33949957 PMC8135033

[31] Kong Y Posada-Quintero HF Chon KH. Sensitive physiological indices of pain based on differential characteristics of electrodermal activity. IEEE Trans Biomed Eng. (2021) 68:3122–30. 10.1109/TBME.2021.3065218 33705307 PMC8483589

[32] Tate JA Seaman JB Happ MB. Overcoming barriers to pain assessment: communicating pain information with intubated older adults. Geriatr Nurs (New York, NY). (2012) 33:310–3. 10.1016/j.gerinurse.2012.06.004 PMC3876478 22771298

[33] Clukey BL Weyant RA Roberts M Henderson A. Discovery of unexpected pain in intubated, sedated patients. Am J Crit Care. (2014) 23:216–20. 10.4037/ajcc2014943 24786809

[34] Thiam P Bellmann P Kestler HA Schwenker F. Exploring deep physiological models for nociceptive pain recognition. Sensors. (2019) 19:4503. 10.3390/s19204503 31627305 PMC6833075

[35] Pouromran F Radhakrishnan S Kamarthi S. Exploration of physiological sensors, features,, machine learning models for pain intensity estimation. PLoS ONE. (2021) 16:e0254108. 10.1371/journal.pone.0254108 34242325 PMC8270203

[36] Othman E Werner P Saxen F Fiedler M-A Al-Hamadi A. An automatic system for continuous pain intensity monitoring based on analyzing data from uni-, bi-, and multi-modality. Sensors. (2022) 22:4992. 10.3390/s22134992 35808487 PMC9269799

[37] Fernandez Rojas R Brown N Waddington G Goecke R. A systematic review of neurophysiological sensing for the assessment of acute pain. npj Digit Med. (2023) 6:76. 10.1038/s41746-023-00810-1 37100924 PMC10133304

[38] Burton AR Fazalbhoy A Macefield VG. Sympathetic responses to noxious stimulation of muscle and skin. Front Neurol. (2016) 7:109. 10.3389/fneur.2016.00109 27445972 PMC4927631

[39] Subramanian S Barbieri R Brown EN. Point process temporal structure characterizes electrodermal activity. Proc Natl Acad Sci. (2020) 117:26422–8. 10.1073/pnas.2004403117 33008878 PMC7584910

[40] Shi Y Liu Z Zhang S Li Q Guo S Yang J, et al. Brain network response to acupuncture stimuli in experimental acute low back pain: an fMRI study. Evidence-Based Complement Altern Med. (2015) 2015. 10.1155/2015/210120 PMC4487721 26161117

[41] Fernandez Rojas R Liao M Romero J Huang X Ou K-L. Cortical network response to acupuncture and the effect of the Hegu point: an fNIRS study. Sensors. (2019) 19:394. 10.3390/s19020394 30669377 PMC6359459

[42] Rojas RF Romero J Lopez-Aparicio J Ou K-L. Pain assessment based on fNIRS using Bi-LSTM RNNs. In *2021 10th International IEEE/EMBS Conference on Neural Engineering (NER)*. IEEE (2021). p. 399–402.

